# THE PANDEMIC AND DIGITAL DIVIDE FOR OLDER ADULTS: LONGITUDINAL ANALYSIS FROM THE NATIONAL HEALTH AGING TRENDS STUDY

**DOI:** 10.1093/geroni/igae098.2341

**Published:** 2024-12-31

**Authors:** BoRin Kim, Joonhyeog Park, Joonyoung Cho, Sojung Park

**Affiliations:** University of New Hampshire, Durham, New Hampshire, United States; School of Social Policy and Practice, University of Pennsylvania, Philadelphia, Pennsylvania, United States; University of Hawaii, Hawaii, United States; Washington University in St. Louis, St. Louis, Missouri, United States

## Abstract

Older adults have been disadvantaged in utilizing information and communication technologies (ICTs). The pandemic has underscored the importance of ICTs in facilitating remote activities such as social connectivity, banking, and shopping. This study explored transitions of ICT use among older adults and investigated the impact of the pandemic on the digital divide. Data came from five waves of the National Health and Aging Trend Study (2018~2022), and our sample included respondents over 70 years at the baseline (N=1,717). Latent transition profile analysis was used to identify distinct subgroups of ICT use and to analyze the change and stability of subgroups based on five ICT use measures: emails, online search, online grocery shopping, Internet banking, and social media. Multinominal regression analyses were used to examine the factors impacting the transitions of ICT use. Four distinct clusters were identified: minimal, email-only, moderate, and diverse/heavy users. The largest subgroup was minimal users (42~45%), followed by diverse/heavy users (25~28%). More than 90% of older adults in these two groups stayed in the same group over time, which shows a significant digital divide. Email-only users were characterized by a stronger tendency toward moderate users (transition probabilities=20~27.5%). While socioeconomic status was a significant predictor of the digital divide before the pandemic, social support was found to be critical to helping older adults use more diverse and frequent ICTs after the pandemic. Our empirical findings provide evidence of exacerbating the digital divide during the pandemic and highlight the importance of social support in reducing the digital divide.

